# Reducing Social Isolation and Improving Nutrition Through Intergenerational Experiential Learning

**DOI:** 10.1093/geroni/igaa057.1744

**Published:** 2020-12-16

**Authors:** Jill Naar

**Affiliations:** Appalachian State University, Boone, North Carolina, United States

## Abstract

This presentation will focus on two projects that aim to inform and educate communities through community based intergenerational programs. An additional link is collaborating with community partner staff on the potential to improve health outcomes with the integration of generations in programming. Through inter-professional collaborations, these projects focus on two areas: reducing social isolation and loneliness among rural older adults through an intergenerational technology program with university students and modifying the implementation of an evidence-based preschool nutrition program for an intergenerational setting. This presentation will focus on the benefits of using community based intergenerational programs and experiential learning opportunities. Aims of this presentation are to highlight how issues related to social isolation and loneliness can be mitigated by incorporating experiential opportunities for university students while simultaneously supporting university students’ preparation for engaging in an intergenerational workforce.

